# Prevalence and determinants of academic burnout among undergraduates in a traditional Chinese medicine university: a cross-sectional study

**DOI:** 10.3389/fpsyg.2026.1799611

**Published:** 2026-06-17

**Authors:** Jie Zhan, Jiagen Xiang, Yiming Zhang, Xingang Zhao, Ke Zhang, Haining Ou, Lechang Zhan, Aiyun He

**Affiliations:** 1 The Second Clinical Medical College, Guangzhou University of Chinese Medicine, Guangzhou, China; 2 Department of Rehabilitation, Guangdong Provincial Hospital of Chinese Medicine, Guangzhou, China; 3 Medical College of Acu-Moxi and Rehabilitation, Guangzhou University of Chinese Medicine, Guangzhou, China; 4 School of Nursing, Guangzhou University of Chinese Medicine, Guangzhou, China; 5 School of Basic Medical Sciences, Guangzhou University of Chinese Medicine, Guangzhou, China

**Keywords:** academic burnout, cross-sectional study, perceived school climate, traditional Chinese medicine, undergraduates

## Abstract

**Background:**

Academic burnout (AB), characterized by emotional exhaustion (EE), cynicism (CY), and reduced academic efficacy (AE) in academic settings, has become a growing concern in higher education. Students in traditional Chinese medicine (TCM) universities face unique challenges due to the demanding curriculum, which integrates both TCM and modern medical knowledge, along with high clinical practice expectations. However, limited data exist on the prevalence and determinants of AB in this population. This study aimed to determine the prevalence and identify the determinants of AB among undergraduates at Guangzhou University of Chinese Medicine (GZUCM).

**Methods:**

A cross-sectional study was conducted from 1 March to 31 August 2025. Using convenience sampling, a self-administered questionnaire was distributed to undergraduates at GZUCM. The questionnaire collected demographic information, academic-related characteristics, health-related behaviors, family background, perceived school climate (assessed with the revised Perceived School Climate Scale), and AB (using the the Chinese version of the Maslach Burnout Inventory-Student Survey, MBI-SS). Data were analyzed using SPSSAU (version 26.0). Descriptive statistics were used to summarize participant characteristics. Differences in AB prevalence across characteristics were assessed using Mann–Whitney U test, Chi-square test, or Fisher’s exact test. Spearman’s correlation analyzed relationships between AB and perceived school climate dimensions. Binary logistic regression (backward LR) identified independent predictors, with adjusted odds ratios (aOR) and 95% confidence intervals (CI) reported.

**Results:**

Out of 747 invited undergraduates, 666 completed the questionnaire (response rate 89.29%). The median age was 21 (IQR: 19, 21) years. The median (IQR) scores on the MBI-SS subscales were 17 (12, 20) for EE, 9 (5, 13) for CY, and 25 (22, 29) for AE. The overall prevalence of AB was 20.87%. Among the burnout subdimensions, high EE was present in 63.66% of undergraduates, high CY in 67.57%, and low AE in 37.24%. Factors significantly associated with AB included religious affiliation (aOR = 5.56, 95% CI: 1.93–15.87, *p* = 0.001), pre-university residence (aOR = 0.60, 95% CI: 0.39–0.94, *p* = 0.027), and regularity of daily routine (aOR = 2.03, 95% CI: 1.32–3.12, *p* = 0.001). Previous semester class ranking was also correlated with higher AB risk. For each one-point increase in the teacher support score, the adjusted odds of AB decreased by 22% (aOR = 0.78, 95% CI: 0.73–0.84, *p* < 0.001).

**Conclusion:**

This study reveals a notable prevalence (20.87%) of AB among TCM undergraduates, with independent determinants including religious affiliation, pre-university urban residence, irregular daily routine, lower academic ranking, and reduced teacher support. These findings highlight that burnout arises from an interplay of demographic, behavioral, academic, and environmental factors. To address this, we propose four actionable strategies: (a) training faculty to provide supportive mentorship and growth-oriented feedback; (b) integrating mandatory courses on time management and sleep hygiene into the core curriculum; (c) implementing targeted support programs for students with lower academic rankings and those from religious minority backgrounds; and (d) establishing cross-year peer mentoring initiatives.

**Clinical trial registration:**

https://www.chictr.org.cn/ identifier ChiCTR2300070050.

## Background

Academic burnout (AB) among university students has emerged as a significant issue in public health and educational psychology (Ntumi et al., 2025). This psychological syndrome results from chronic academic stress and is characterized by three dimensions: emotional exhaustion (EE), cynicism (CY), and reduced academic efficacy (AE) (Schaufeli et al., 2002). Globally, the prevalence estimates of AB vary widely from 7.0 to 75.2% among medical students, depending on measurement tools and cut-off criteria (Erschens et al., 2019; Frajerman et al., 2019). In China, studies have reported AB rates of 20.5% among general university students (Liu et al., 2023) and 45.9% among medical students (Li et al., 2021). In contrast to ordinary stress, AB can detrimentally impact mental health and quality of life, increase the risk of anxiety, depression, and sleep disorders, and may lead to academic disengagement, cognitive impairments, and unprofessional conduct, thereby potentially influencing long-term career trajectories (Schaufeli et al., 2002; Njim et al., 2019).

Medical students are especially susceptible to AB due to the intensive demands of coursework, clinical internships, high-pressure environments, and career uncertainty (Lu et al., 2024). Research suggests that students in Traditional Chinese Medicine (TCM) institutions may face even higher risks (Zhang et al., 2022). This disparity points to unique contributing factors within the TCM higher education system that extend beyond the universal stressors of medical education. A key challenge for TCM students lies in their dual pedagogical burden, as they are required to master not only the foundational TCM courses (including the classics of TCM, courses of acupuncture and moxibustion, and clinical practice) but also a comprehensive body of modern medical knowledge (Xue and Jian-Ping, 2016). For example, a typical day’s timetable for a TCM student might include detailed study sessions in ancient TCM texts and methodologies in the mornings, followed by afternoons filled with biomedicine lectures and practical laboratory work. Also, this blend of traditional and modern medical education demands adaptability and resilience. Balancing these two domains imposes significant cognitive demands and necessitates prolonged practice, resulting in a distinct stress profile. This dual-curriculum challenge is relatively unique to TCM education in China. In comparison, medical students in most Western countries follow a predominantly biomedicine-centered curriculum without a parallel traditional medical system. However, some parallels exist: students in complementary and alternative medicine (CAM) programs in countries such as India (Ayurveda), Korea (traditional Korean medicine), and Japan (Kampo) face similar dual training demands (Navghare et al., 2025). Thus, while the specific content differs, the phenomenon of integrating two distinct medical knowledge systems is not exclusive to China. Understanding how TCM students cope with this dual burden may offer insights for CAM education globally. Therefore, examining AB among TCM students is both timely and necessary.

Previous research has explored individual determinants of AB, including personality traits, coping strategies, and sociodemographic characteristics, yet findings remain inconsistent (Kashefian-Naeeini et al., 2025; Aguayo et al., 2019). Increasingly, broader ecological factors, such as family and school environments, are recognized as influential. For instance, family-related variables, including parental education and socioeconomic status, may affect burnout risk by shaping parental involvement, access to resources, and psychological security (Maynard et al., 2014; Ali et al., 2020). Additionally, school climate plays a critical role, where supportive relationships and autonomy can mitigate academic stress and foster student development (Manikandan and Sujisha, 2014; Podiya et al., 2025). These elements highlight the growing acknowledgment of the multifaceted nature of burnout, where individual, family, and wider environmental contexts interact. Nevertheless, limited research in Chinese higher education, particularly within TCM universities, has systematically examined the combined effects of these factors on AB. Specifically, three gaps remain: (a) most existing studies have focused on a single level of factors (e.g., individual personality or academic stress) rather than integrating individual, family, and environmental influences; (b) few studies have applied validated measures of perceived school climate (teacher support, peer support, autonomy) to TCM student populations; and (c) the unique dual-curriculum challenge in TCM education has not been adequately investigated as a contextual moderator of AB risk. These gaps constrain comprehensive understanding and the formulation of effective, multi-level interventions.

Therefor, this study aims to: (a) estimate the overall prevalence of AB among undergraduates at Guangzhou University of Chinese Medicine (GZUCM); (b) systematically examine the influence of multilevel factors, including demographic characteristics, health-related behaviors, family background, and perceived school climate, on AB; and (c) identify independent risk and protective factors for AB within this student population.

## Methods

### Ethics approval and consent to participate

Ethical approval for this study was conducted by the Ethics Committee of Guangdong Provincial Hospital of Chinese Medicine (No. ZM2023-080). The study was conducted in accordance with the ethical principles outlined in the Declaration of Helsinki. All participants were informed about the purpose of the study, assured of confidentiality, and provided an online informed consent prior to participation. Participation was voluntary, and respondents could withdraw at any time without consequence.

### Study design and study setting

This cross-sectional study was conducted at GZUCM in China from 1 March to 31 August 2025. As one of China’s first four institutions for higher TCM education, GZUCM is a national “Double First-Class” university with significant representation in TCM higher education. Founded in 1924, it boasts a rich heritage in TCM education, with disciplines centered on TCM (including a national “Double First-Class” TCM discipline). The university enrolls over 12,000 full-time undergraduates, providing an ample sampling frame. Selecting participants from this university ensures that AB is investigated within a representative TCM higher education context.

### Study population

Participants were recruited via convenience sampling from the target undergraduate population, with eligibility determined by the following criteria:

Inclusion Criteria: (a) Currently enrolled full-time undergraduate students; (b) Aged 16–35 years; (c) Be able to use a smartphone or the internet competently; (d) No major life events in the past 3 months; (e) Willingness to provide accurate health and academic information; and (f) Provision of voluntary informed consent.

Exclusion Criteria: (a) Current or past three-month use of anti-anxiety/antidepressant medications or participation in psychotherapy; (b) Conditions precluding questionnaire completion (e.g., severe visual impairment); or (c) Other investigator-determined ineligibility.

Eligible participants were informed of the study’s objectives and the principles of voluntariness and anonymity.

### Study sample

One of the primary objectives of this study was to estimate the prevalence of AB within the target undergraduate population. Sample size was calculated using the standard formula for estimating proportions in cross-sectional studies: n = [Z^2^ × P × (1-P)] / d^2^, where n is the required sample size, Z is the statistic corresponding to the confidence level (Z = 1.96 for *α* = 0.05), P is the expected prevalence, and d is the margin of error (0.05) (Charan and Biswas, 2013). Due to limited prior prevalence data on AB (using MBI-SS criteria) in similar populations, we used a standard conservative approach (*p* = 0.5) in sample size calculation, as it maximizes the required sample size and ensures adequate statistical power for detecting associations (Hajian-Tilaki, 2011; Charan and Biswas, 2013). And the minimum required sample size was calculated as 384. Accounting for an 90% valid response rate, the target sample size was adjusted to 427. This anticipated response rate is consistent with previous online surveys conducted among Chinese university students (Liu et al., 2023). Following questionnaire distribution, collection, and rigorous quality control procedures, 666 valid questionnaires were included in the analysis. This sample size substantially exceeded the minimum requirement and provided adequate statistical power.

### Data collection

Online data collection was conducted via “Wenjuanxing,”^1^ a widely used professional online survey platform in China. A unique electronic questionnaire link and a corresponding QR code were generated and distributed to potential participants, who could access and complete the survey by scanning the code. Participants completed surveys under supervised conditions in designated on-campus locations, organized by their respective classes with assistance from student counselors.

### Data quality control

A comprehensive quality control protocol was implemented throughout the study. Investigator training included standardized instruction on study objectives, questionnaire administration, instructional scripts, and privacy and confidentiality protocols. During offline sessions, trained investigators provided consistent explanations, addressed real-time questions, and emphasized anonymity to promote independent and honest responses. Collected questionnaires were immediately reviewed for logical consistency and completeness. Invalid questionnaires were excluded based on predefined criteria, such as incomplete responses (missing data for more than 20% of key variables) or evidence of patterned or illogical responses (such as straight-line answers or selecting the same option for all items). These measures were designed to maximize the authenticity and validity of the data for analysis.

### Survey development

#### General information questionnaire

Structured questionnaires collected: (a) Sociodemographic characteristics: age, gender, only-child status (yes/no), ethnicity (Han/other), religious affiliation (yes/no), and pre-university residence (urban/rural); (b) Academic-related characteristics: grade, major, previous semester class ranking, and experience in student organization leadership (yes/no); (c) Health-related behaviors: smoking status (yes/no), alcohol consumption (yes/no), regularity of diet (yes/no), regularity of daily routine (yes/no), and history of diagnosed mental disorders (yes/no); (d) Family background: family monthly income and parental highest education level.

#### Academic burnout

AB was assessed using the Chinese version of the Maslach Burnout Inventory-Student Survey (MBI-SS) (Hu and Schaufeli, 2009). This 15-item instrument measures three dimensions: EE (five items), CY (four items), and AE (six items). Responses are recorded on a 7-point Likert scale ranging from 0 (“never”) to 6 (“every day”). Following established cut-off points, scores for each dimension were categorized as low, moderate, or high: EE (low: 0–9; moderate: 10–14; high: ≥15), CY (low: 0–1; moderate: 2–6; high: ≥7), and AE (low: 0–22; moderate: 23–27; high: ≥28) (Campos and Maroco, 2012). Consistent with the standard diagnostic criteria, participants exhibiting a profile of ‘high EE, high CY, and low AE’ were classified as having AB (Campos and Maroco, 2012). In this study, the scale demonstrated acceptable to good internal consistency, with Cronbach’s alpha coefficients of 0.747 for the total MBI-SS, 0.833 for EE, 0.768 for CY, and 0.854 for AE (Supplementary Table 1).

**Table 1:** Baseline characteristics of students who participated in study.

Variables	n (%)/M (P25–P75)
Demographics	
Age (years)	21 (19.00–21.00)
Gender	
Female	130 (19.52%)
Male	536 (80.48%)
The only child	
Yes	128 (19.22%)
No	538 (80.78%)
Ethnicity	
Han	624 (93.69%)
Other	42 (6.31%)
Religious affiliation	
Yes	20 (3%)
No	646 (97%)
Pre-university residence	
Urban	399 (59.91%)
Rural	267 (40.09%)
Health-related behaviors and status	
Smoking status	
Yes	6 (0.9%)
No	660 (99.1%)
Alcohol consumption	
Yes	13 (1.95%)
No	653 (98.05%)
Regularity of diet	
Yes	483 (72.52%)
No	183 (27.48%)
Regularity of daily routine	
Yes	380 (57.06%)
No	286 (42.94%)
History of mental disorder	
Yes	19 (2.85%)
No	647 (97.15%)
Academic-related characteristics	
Grade	
Lower	338 (50.76%)
1	169 (25.38%)
2	169 (25.38%)
Upper	328 (49.24%)
3	161 (24.17%)
4	153 (22.97%)
5	14 (2.1%)
Major	
Nursing	398 (59.76%)
Other	268 (40.24%)
Previous semester class ranking	
Top 5%	61 (9.16%)
Top 6–20%	177 (26.58%)
Top 21–50%	206 (30.93%)
Top 51–80%	166 (24.92%)
Bottom 20%	56 (8.41%)
Leadership experience in student organizations	
Yes	548 (82.28%)
No	118 (17.72%)
Family background	
Family monthly income (CNY)	
4,000 and below	145 (21.77%)
4,001–8,000	242 (36.34%)
8,001 and above	279 (41.89%)
Father’s highest educational attainment	
Primary school or below	86 (12.91%)
Junior high school	211 (31.68%)
High school	189 (28.38%)
Undergraduate and above	180 (27.03%)
Mother’s highest educational attainment	
Primary school or below	136 (20.42%)
Junior high school	235 (35.29%)
High school	144 (21.62%)
Undergraduate and above	151 (22.67%)
Academic burnout	
Emotional exhaustion	17 (12.00–20.00)
Cynicism	9 (5.00–13.00)
Academic efficacy	25 (22.00–29.00)
Perceived school climate	
Teacher support	19 (16.00–20.00)
Student - student support	45 (41.00–47.00)
Opportunities for autonomy	12 (10.00–15.00)
Perceived School Climate Scale	76 (69.00–81.00)

#### Perceived school climate

The perceived school climate was assessed using the revised Perceived School Climate Scale (PSCS) by Jia et al. (2009). This instrument comprises 25 items across three dimensions: Teacher Support (TS, seven items), Student–Student Support (SSS, 13 items), and Opportunities for Autonomy (OFA, five items). Responses were recorded on a 4-point Likert scale, with seven reverse-scored items (2, 3, 10, 13, 18, 20, and 23). Higher scores indicated a more positive perceived school climate. In the present study, the scale demonstrated good internal consistency, with Cronbach’s alpha coefficients of 0.910 for the total PSCS, 0.879 for the TS subscale, 0.867 for the SSS subscale, and 0.897 for the OFA subscale (Supplementary Table 1).

### Statistical analysis

Data were processed and analyzed using SPSSAU (version 26.0, https://www.spssau.com). Normally distributed continuous variables were presented as mean ± standard deviation; non-normal variables as median (interquartile range, IQR). Categorical variables were described as frequencies (percentages). Differences in academic burnout prevalence across characteristics were assessed using the Mann–Whitney U test, Chi-square test, or Fisher’s exact test, as appropriate. Spearman’s correlation was used to analyze the relationships between the dimensions of AB and the dimensions of perceived school climate. Binary logistic regression (backward LR method) was performed with AB as the dependent variable, including all independent variables with *p* < 0.05 in univariate analysis to calculate adjusted odds ratio (aOR) and 95% confidence intervals (CIs). Two-sided tests were used, with *p* < 0.05 considered statistically significant.

## Results

### Sample characteristics

**Figure 1:**
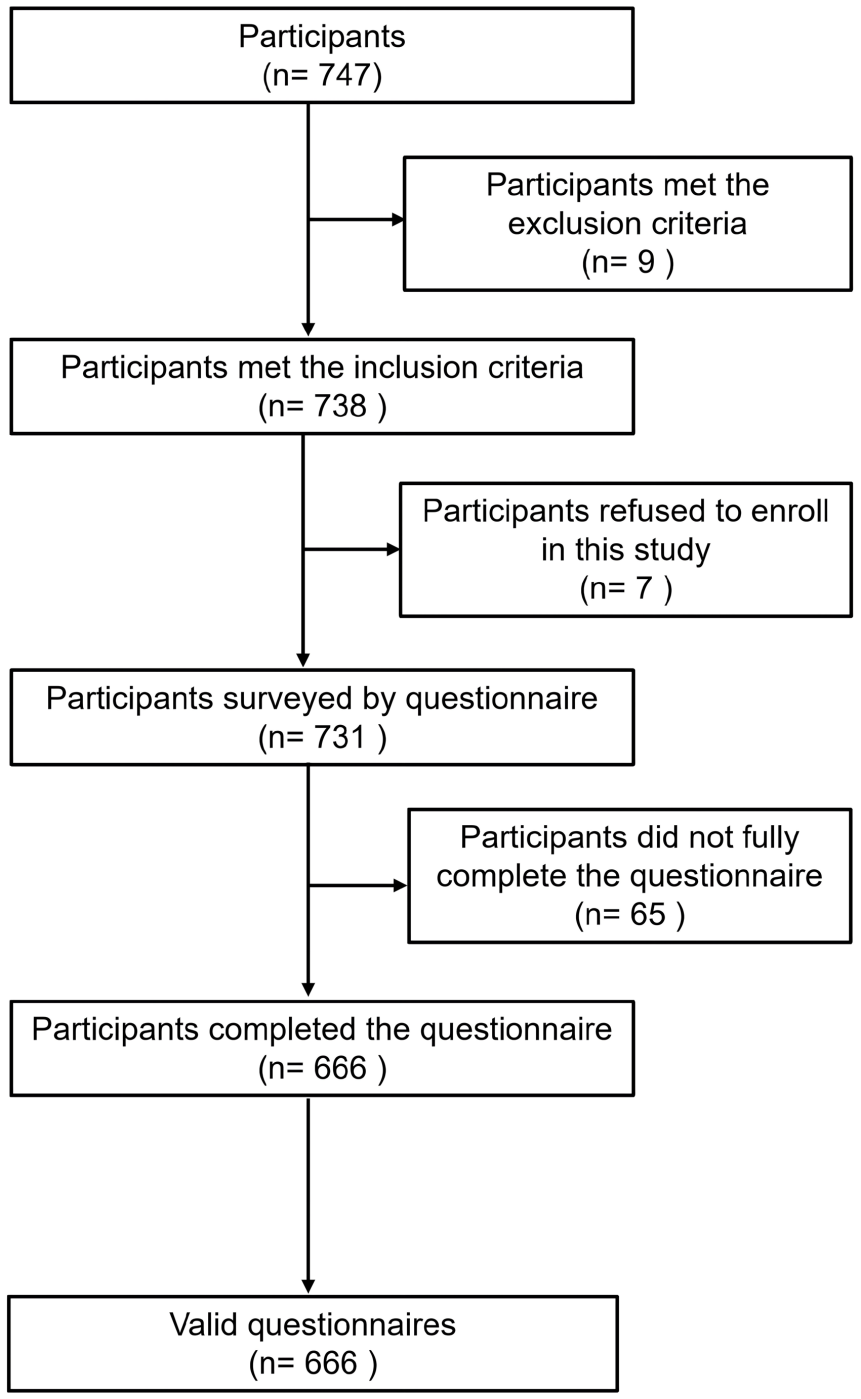
Research flow diagram.

A total of 747 undergraduates were invited to participate in this survey, of whom 738 met the inclusion criteria. After being informed of the study details, seven undergraduates declined to participate, primarily due to a lack of interest. Additionally, 65 questionnaires were excluded from the final analysis because more than 20% of the key variable data were missing. Ultimately, 666 valid questionnaires were included in the statistical analysis, corresponding to an effective response rate of 89.29%. The detailed participant inclusion process is visually depicted in Figure 1.

Among the 666 valid participants, the median age was 21 (IQR: 19.00, 21.00) years. Regarding gender distribution, male students accounted for 80.48% of the sample. The proportion of only-child students was 19.22%, while Han Chinese accounted for the majority at 93.69%. Regarding religious affiliation, 97.00% of participants reported having none. Regarding pre-university residential background, 59.91% of the students had resided in urban areas before enrolling in university (Table **1**).

Regarding health-related behaviors, the vast majority of participants reported being non-smokers (99.10%) and non-drinkers (98.05%). Specifically, 72.52% of the students maintained regular dietary habits, and 57.06% reported having a regular daily routine. Notably, only 2.85% of the participants had a history of mental disorders (Table **1**).

Regarding academic characteristics, the sample was nearly equally divided between lower-year (defined as first- and second-year students) (50.76%) and upper-year (defined as third-, fourth-, and fifth-year students) (49.24%) students. Nursing majors formed the largest group, accounting for 59.76% of the total participants. Leadership experience in student organizations was common, with 82.28% of the students reporting such expertise. Regarding socioeconomic status, 41.89% of participants reported that their family’s average monthly income exceeded 8,000 Chinese Yuan (CNY) (Table **1**).

### Prevalence of academic burnout

**Figure 2:**
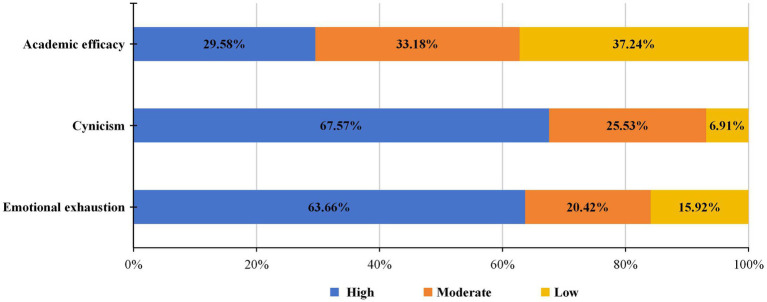
Prevalence of three academic burnout domains among the study participants.

**Figure 3:**
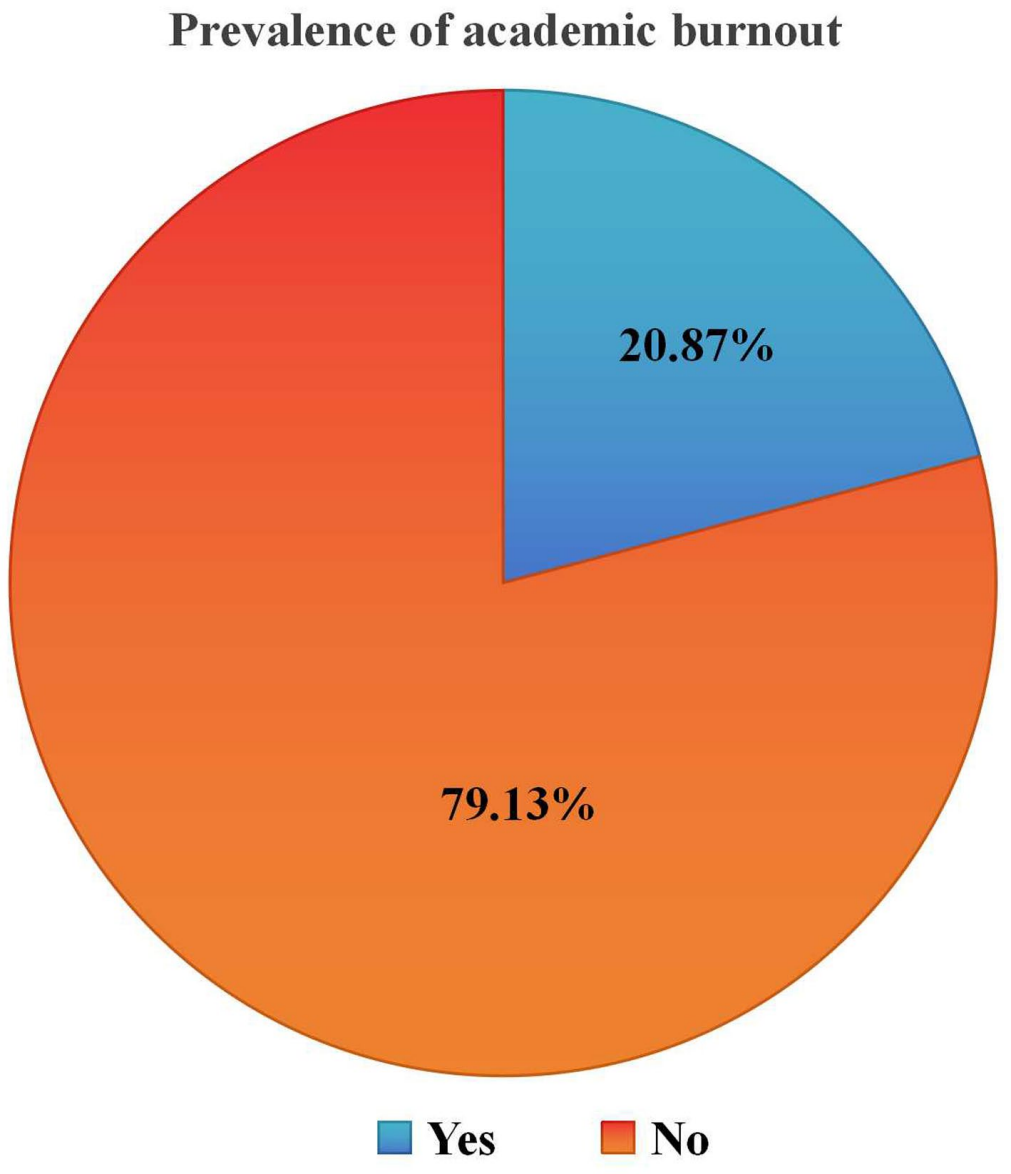
Prevalence of academic burnout among the study participants.

Among the 666 valid survey respondents, the detection rates of AB dimensions were as follows: 63.66% reported high EE, 67.57% reported high CY, and 37.24% reported low AE (Figure 2). In this study, consistent with the standard three-dimensional model of burnout, individuals who simultaneously met the criteria for high EE (≥ 15), high CY (≥ 7), and low AE (≤ 22) were classified as having AB (Campos and Maroco, 2012). The overall prevalence of AB in the sample was 20.87% (Figure 3).

Stratified analysis by socio-demographic characteristics revealed no significant differences in AB prevalence between genders or between only-child and non-only-child groups. However, notable disparities were observed across other variables. Specifically, Han Chinese undergraduates exhibited a significantly lower prevalence of AB (19.71%) than students from different ethnic groups (38.10%, *p* < 0.01). Additionally, students with religious affiliation had a markedly higher prevalence (55.00%) than those without (19.81%, *p* < 0.01). It is also worth noting that undergraduates who had resided in urban areas before university enrollment had a significantly higher prevalence of AB (23.81%) than their rural counterparts (16.48%) (Table 2).

**Table 2:** Prevalence of academic burnout by different characteristics of the study participants.

Variables	Academic burnout		Prevalence of academic burnout (%)	Statistic	*P*
	No (*n* = 527)	Yes (*n* = 139)			
Demographics					
Age, M (P25–P75)	21 (19.00–21.00)	20 (19.00–21.00)	-	Z = −1.61	0.11
Gender, *n* (%)					
Female	98 (18.60)	32 (23.02)	24.62	χ^2^ = 1.37	0.24
Male	429 (81.40)	107 (76.98)	19.96		
The only child, *n* (%)					
Yes	95 (18.03)	33 (23.74)	25.78	χ^2^ = 2.31	0.13
No	432 (81.97)	106 (76.26)	19.70		
Ethnicity, *n* (%)					
Han	501 (95.07)	123 (88.49)	19.71	χ^2^ = 8.05	<0.01
Other	26 (4.93)	16 (11.51)	38.10		
Religious affiliation, *n* (%)					
No	518 (98.29)	128 (92.09)	19.81	χ^2^ = 14.54	<0.01
Yes	9 (1.71)	11 (7.91)	55.00		
Pre-university residence, *n* (%)					
Urban	304 (57.69)	95 (68.35)	23.81	χ^2^ = 5.20	0.02
Rural	223 (42.31)	44 (31.65)	16.48		
Health-related behaviors and status					
Smoking status, *n* (%)					
Smokers	3 (0.57)	3 (2.16)	50.00	χ^2^ = 3.11	0.08
Non-smokers	524 (99.43)	136 (97.84)	20.61		
Alcohol consumption, *n* (%)					
Drinkers	9 (1.71)	4 (2.88)	30.77	χ^2^ = 0.79	0.38
Non-drinkers	518 (98.29)	135 (97.12)	20.67		
Regularity of diet, *n* (%)					
Yes	401 (76.09)	82 (58.99)	16.98	χ^2^ = 16.14	<0.01
No	126 (23.91)	57 (41.01)	31.15		
Regularity of daily routine, *n* (%)					
Yes	323 (61.29)	57 (41.01)	15.00	χ^2^ = 18.47	<0.01
No	204 (38.71)	82 (58.99)	28.67		
History of mental disorder, n(%)					
Yes	10 (1.90)	9 (6.47)	47.37	χ^2^ = 8.32	<0.01
No	517 (98.10)	130 (93.53)	20.09		
Academic-related characteristics					
Grade, *n* (%)					
Lower	256 (48.58)	82 (58.99)	24.26	χ^2^ = 4.77	0.03
Upper	271 (51.42)	57 (41.01)	17.38		
Major, *n* (%)					
Nursing	323 (61.29)	75 (53.96)	18.84	χ^2^ = 2.46	0.12
Other	204 (38.71)	64 (46.04)	23.88		
Previous semester class ranking, *n* (%)					
Top 5%	54 (10.25)	7 (5.04)	11.48	χ^2^ = 28.67	<0.01
Top 6–20%	155 (29.41)	22 (15.83)	12.43		
Top 21–50%	164 (31.12)	42 (30.22)	20.39		
Top 51–80%	121 (22.96)	45 (32.37)	27.11		
Bottom 20%	33 (6.26)	23 (16.55)	41.07		
Leadership experience in student organizations, *n* (%)					
Yes	437 (82.92)	111 (79.86)	20.26	χ^2^ = 0.71	0.40
No	90 (17.08)	28 (20.14)	23.73		
Family background					
Family monthly income (CNY), *n* (%)					
4,000 and below	117 (22.20)	28 (20.14)	19.31	χ^2^ = 0.86	0.65
4,001–8,000	194 (36.81)	48 (34.53)	19.83		
8,001 and above	216 (40.99)	63 (45.32)	22.58		
Father’s highest educational attainment, *n* (%)					
Primary school or below	65 (12.33)	21 (15.11)	24.42	χ^2^ = 1.80	0.61
Junior high school	169 (32.07)	42 (30.22)	19.91		
High school	154 (29.22)	35 (25.18)	18.52		
Undergraduate and above	139 (26.38)	41 (29.50)	22.78		
Mother’s highest educational attainment, n(%)					
Primary school or below	108 (20.49)	28 (20.14)	20.59	χ^2^ = 0.73	0.87
Junior high school	189 (35.86)	46 (33.09)	19.57		
High school	114 (21.63)	30 (21.58)	20.83		
Undergraduate and above	116 (22.01)	35 (25.18)	23.18		
Perceived school climate, M (P25–P75)					
Teacher support	19 (17.00–21.00)	16 (14.00–18.00)	-	Z = −8.92	<0.01
Student—student support	45 (42.00, 48.00)	43 (40.00–45.00)	-	Z = −5.43	<0.01
Opportunities for autonomy	13 (10.00–15.00)	11 (10.00–13.00)	-	Z = −4.92	<0.01
Perceived School Climate Scale	77 (71.00–82.00)	70 (64.00–75.50)	-	Z = −7.65	<0.01

In the analysis of health-related behaviors and status, no significant differences in AB prevalence were found between smokers and non-smokers, or between alcohol drinkers and non-drinkers. However, certain lifestyle factors demonstrated significant protective effects. Specifically, the prevalence of AB was significantly lower among students with regular dietary habits (16.98%) and those with regular daily routines (15.00%) (both *p* < 0.01). In contrast, participants with a history of mental disorders showed a substantially higher prevalence of AB (47.37%) (Table 2).

Regarding academic characteristics, lower-year students had a higher prevalence of AB than upper-year students (24.26% vs. 17.38%, *p* = 0.03). The prevalence among nursing majors was comparable to that of students from other majors. Notably, a higher academic performance ranking within the class was inversely associated with the risk of AB (*p* < 0.01). No significant difference in burnout rates was observed between students with and without student organization leadership experience (20.26% vs. 23.73%, *p* = 0.40). In terms of family background, neither family monthly income nor parents’ highest educational level showed a significant association with the prevalence of AB (both *p* > 0.05) (Table 2).

### Correlations between subscales of academic burnout and perceived school climate

Among the 666 participants, the median (IQR) scores for the MBI-SS subscales were as follows: EE = 17 (12.00, 20.00), CY = 9 (5.00, 13.00), and AE = 25 (22.00, 29.00). For the PSCS subscales, the median (IQR) scores were: TS = 19 (16.00, 20.00), SSS = 45 (41.00, 47.00), and OFA = 12 (10.00, 15.00). The total PSCS score had a median (IQR) of 76 (69.00, 81.00) (Table 1).

Spearman correlation analysis revealed that TS scores (r = −0.308, *p* < 0.01), SSS scores (r = −0.183, *p* < 0.01), and OFA scores (r = −0.213, *p* < 0.01) were all negatively correlated with EE scores. Similarly, TS scores (r = −0.384, *p* < 0.01), SSS scores (r = −0.349, *p* < 0.01), and OFA scores (r = −0.217, *p* < 0.01) exhibited negative correlations with CY scores. In contrast, TS scores (r = 0.476, *p* < 0.01), SSS scores (r = 0.336, *p* < 0.01), and OFA scores (r = 0.283, *p* < 0.01) were positively correlated with AE scores. Detailed correlation results are presented in Table 3.

**Table 3:** Correlations between subscales of academic burnout and perceived school climate of the study participants.

Variables	EE	CY	AE	TS	SSS	OFA	PSCS
EE	1						
CY	0.687^**^	1					
AE	−0.299^**^	−0.396^**^	1				
TS	−0.308^**^	−0.384^**^	0.476^**^	1			
SSS	−0.183^**^	−0.349^**^	0.336^**^	0.443^**^	1		
OFA	−0.213^**^	−0.217^**^	0.283^**^	0.499^**^	0.387^**^	1	
PSCS	−0.276^**^	−0.399^**^	0.446^**^	0.771^**^	0.823^**^	0.736^**^	1

AE, academic efficacy; CY, cynicism; EE, emotional exhaustion; OFA, opportunities for autonomy; PSCS, Perceived School Climate Scale; SSS, student - student support; TS, teacher support. Spearman’s correlation test, **p <0.01.

### Factors associated with academic burnout

Univariate logistic regression analysis identified several variables significantly associated with AB (*p* < 0.05), including ethnicity (Han or other), religious affiliation (yes/no), pre-university residence (urban/rural), regularity of diet (yes/no), regularity of daily routine (yes/no), history of mental disorders (yes/no), grade (lower/upper), previous semester’s class ranking, TS score, SSS score, OFA score, and PSCS score. These twelve variables were subsequently included in the multicollinearity analysis. A variable was considered to have collinearity issues if the absolute value of the correlation coefficient between two variables > 0.8, or if the variance inflation factor value was > 5, or if the tolerance value was < 0.2. The results indicated that PSCS exhibited collinearity problems, and this variable was removed from the model. The remaining variables were incorporated into the multivariable binary logistic regression analysis.

Based on the multivariable binary logistic regression analysis presented in Table 4, five independent factors were identified as significant predictors of AB (all *p* < 0.05), after adjusting for all other variables in the model (Nagelkerke R-squared = 0.285; Likelihood ratio test: χ^2^ = 134.379, df = 9, *p* < 0.001; Hosmer-Lemeshow goodness-of-fit test: χ^2^ = 7.248, df = 8, *p* = 0.510). Specifically, compared to students with no religious affiliation, undergraduates with a religious affiliation had significantly higher odds of experiencing AB (aOR = 5.56, 95% CI: 1.93–15.87, *p* = 0.001). Similarly, students who had resided in rural areas before university had lower odds of AB than their urban counterparts (aOR = 0.60, 95% CI: 0.39–0.94, *p* = 0.027). Students reporting an irregular daily routine had more than twice the odds of AB compared to those with a regular routine (aOR = 2.03, 95% CI: 1.32–3.12, *p* = 0.001). Academic performance emerged as a strong predictor: compared to students ranked in the top 5%, those in the top 51–80% (aOR = 2.23, 95% CI: 1.25–3.99, *p* = 0.007) and those in the bottom 20% (aOR = 3.87, 95% CI: 1.85–8.08, *p* < 0.001) had significantly higher odds of AB. Notably, each one-point increase in the TS score was associated with a 22% reduction in the odds of AB (aOR = 0.78, 95% CI: 0.73–0.84, *p* < 0.001).

**Table 4:** Screening out factors associated with academic burnout in study participants via univariate and multivariable binary logistic regression.

Variables	Univariate ^#^					Multivariable ^#*^				
	β	S.E	Z	*P*	OR (95%CI)	β	S.E	Z	*P*	Adjusted OR (95% CI)
Age	−0.12	0.07	−1.66	0.097	0.89 (0.77 ~ 1.02)					
Gender										
Female					Reference					
Male	−0.27	0.23	−1.17	0.243	0.76 (0.49 ~ 1.20)					
The only child										
Yes					Reference					
No	−0.35	0.23	−1.52	0.130	0.71 (0.45 ~ 1.11)					
Ethnicity										
Han					Reference					Reference
Other	0.92	0.33	2.76	**0.006**	2.51 (1.30 ~ 4.82)	0.73	0.38	1.92	0.055	2.07 (0.99 ~ 4.34)
Religious affiliation										
No					Reference					Reference
Yes	1.60	0.46	3.47	**0.001**	4.95 (2.01 ~ 12.20)	1.71	0.54	3.19	**0.001**	5.56 (1.93 ~ 15.87)
Pre-university residence										
Urban					Reference					Reference
Rural	−0.46	0.20	−2.27	**0.023**	0.63 (0.43 ~ 0.94)	−0.51	0.23	−2.22	**0.027**	0.60 (0.39 ~ 0.94)
Smoking status										
Smokers					Reference					
Non-smokers	−1.35	0.82	−1.64	0.101	0.26 (0.05 ~ 1.30)					
Alcohol consumption										
Drinkers					Reference					
Non-drinkers	−0.53	0.61	−0.88	0.380	0.59 (0.18 ~ 1.93)					
Regularity of diet										
Yes					Reference					
No	0.79	0.20	3.96	**<0.001**	2.21 (1.49 ~ 3.28)					
Regularity of daily routine										
Yes					Reference					Reference
No	0.82	0.19	4.24	**<0.001**	2.28 (1.56 ~ 3.33)	0.71	0.22	3.25	**0.001**	2.03 (1.32 ~ 3.12)
History of mental disorder										
Yes					Reference					
No	−1.28	0.47	−2.71	**0.007**	0.28 (0.11 ~ 0.70)					
Grade										
Lower					Reference					Reference
Upper	−0.42	0.19	−2.18	**0.029**	0.66 (0.45 ~ 0.96)	−0.40	0.22	−1.84	0.066	0.67 (0.44 ~ 1.03)
Major										
Nursing					Reference					
Other	0.30	0.19	1.57	0.117	1.35 (0.93 ~ 1.97)					
Previous semester class ranking										
Top 5%					Reference					Reference
Top 6–20%	0.09	0.46	0.20	0.844	1.10 (0.44 ~ 2.71)	-	-	-	-	-
Top 21–50%	0.68	0.44	1.56	0.120	1.98 (0.84 ~ 4.66)	0.55	0.29	1.88	0.060	1.73 (0.98 ~ 3.08)
Top 51–80%	1.05	0.44	2.41	**0.016**	2.87 (1.22 ~ 6.77)	0.80	0.30	2.72	**0.007**	2.23 (1.25 ~ 3.99)
Bottom 20%	1.68	0.49	3.47	**0.001**	5.38 (2.08 ~ 13.91)	1.35	0.38	3.60	**<0.001**	3.87 (1.85 ~ 8.08)
Leadership experience in student organizations										
Yes					Reference					
No	0.20	0.24	0.84	0.400	1.23 (0.76 ~ 1.97)					
Family monthly income (CNY)										
4,000 and below					Reference					
4,001–8,000	0.03	0.27	0.13	0.900	1.03 (0.62 ~ 1.74)					
8,001 and above	0.20	0.25	0.78	0.437	1.22 (0.74 ~ 2.01)					
Father’s highest educational attainment										
Primary school or below					Reference					
Junior high school	−0.26	0.31	−0.86	0.389	0.77 (0.42 ~ 1.40)					
High school	−0.35	0.31	−1.12	0.261	0.70 (0.38 ~ 1.30)					
Undergraduate and above	−0.09	0.31	−0.30	0.767	0.91 (0.50 ~ 1.67)					
Mother’s highest educational attainment										
Primary school or below					Reference					
Junior high school	−0.06	0.27	−0.24	0.814	0.94 (0.56 ~ 1.59)					
High school	0.02	0.30	0.05	0.960	1.02 (0.57 ~ 1.81)					
Undergraduate and above	0.15	0.29	0.53	0.597	1.16 (0.66 ~ 2.04)					
Teacher support	−0.27	0.03	−8.06	**<0.001**	0.77 (0.72 ~ 0.82)	−0.25	0.03	−7.19	**<0.001**	0.78 (0.73 ~ 0.84)
Student—student support	−0.08	0.02	−4.50	**<0.001**	0.92 (0.89 ~ 0.96)					
Opportunities for autonomy	−0.14	0.03	−4.42	**<0.001**	0.87 (0.82 ~ 0.92)					
Perceived school climate scale	−0.08	0.01	−7.02	**<0.001**	0.93(0.91 ~ 0.95)					

OR, odds ratio; CI, confidence interval. ^#^The dependent variable was the presence of academic burnout (yes/no). ^*^Nagelkerke R-squared = 0.285; Likelihood ratio test: χ^2^ = 134.379, df = 9, *p* < 0.001; Hosmer-Lemeshow test: χ^2^ = 7.248, df = 8, *p* = 0.510.

## Discussion

This study conducted a multi-dimensional analysis of AB among a representative sample of TCM undergraduates. The overall AB prevalence of 20.87% is consistent with but at the lower end of the range reported in previous studies on medical students in China (Chunming et al., 2017). Several factors may explain the relatively lower prevalence observed in this study compared to previous reports. First, differences in measurement tools and cut-off criteria may play a major role. Some prior studies used single-dimension measures (e.g., only emotional exhaustion) or lower cut-off thresholds, resulting in higher reported rates. Our application of the stringent “high EE + high CY + low AE” criterion may yield a more conservative estimate (Campos and Maroco, 2012). Second, the timing of data collection (March–August 2025) occurred during a post-pandemic recovery period when college students reported a significant increase in perceived social support, emotional intelligence, and hope (Mah et al., 2026). Third, GZUCM, as a “Double First-Class” university, may have more robust student support services compared to other institutions. More importantly, the multivariate analysis in this study identified a distinct etiological profile, with five independent predictors: religious affiliation, pre-university urban residence, irregular daily routine, lower academic ranking, and lower perceived teacher support.

### Prevalence of academic burnout

Analysis of AB prevalence across different subgroups reveals distinct patterns that require further interpretation, as they point to specific vulnerabilities and potential mechanisms within the TCM student population. The significantly higher AB prevalence among non-Han ethnic students and those with religious affiliation converges on a common theme: the experience of being a minority in the campus environment. In this sample, where Han ethnicity and non-religious affiliation are predominant, these groups may face acculturative stress or a sense of non-belonging. Acculturative stress refers to the psychological strain experienced when adapting to a dominant culture that differs from one’s own background (Hovey and King, 1996; Berry, 1997). Specific manifestations may include: (a) language or dialect differences that hinder effective communication; (b) cultural value conflicts, such as collectivism vs. individualism; (c) perceived or anticipated discrimination; (d) lack of culturally congruent social support networks; and (e) internal identity conflict between one’s heritage and the mainstream culture. The constant need to navigate a majority culture, potentially coupled with a lack of tailored social or spiritual support systems within the university, can act as a chronic stressor. This aligns with minority stress theory, which posits that perceived discrimination and efforts to reconcile different identity aspects can deplete emotional resources (Meyer, 1995; Meyer, 2003), thereby increasing the risk of AB. This finding underscores the need for inclusive policies and support networks that recognize and address the unique needs of minority student groups. Specific actionable strategies include: (a) establishing a Multicultural Student Center offering culturally sensitive counseling and peer support groups; (b) training mental health professionals in cultural competence to recognize minority stress; (c) creating affinity groups for minority-ethnicity and religious students; (d) incorporating diversity and inclusion modules into freshman orientation; and (e) implementing anonymous reporting systems for discrimination or microaggressions.

Contrary to the assumption that rural students might be more vulnerable due to fewer resources, our data indicated that pre-university urban residence was associated with higher AB risk. This may reflect differences in pre-existing academic socialization. In China, educational achievement is highly valued, and competition is intense, particularly in urban areas (Xiao and Liu, 2025). Urban students often come from highly competitive secondary education systems; upon entering university, they may carry forward internalized high expectations and a chronic “pressure-cooker” mindset, making them more likely to perceive academic demands as overwhelming. In contrast, for some rural students, entering university itself represents a significant achievement and a novel, enriching environment, which may initially buffer against academic cynicism-though this protective effect might diminish over time.

The strong protective effect of maintaining regular daily routines and dietary habits represents one of the most actionable findings. Among university students, insufficient sleep and unhealthy dietary practices are frequently associated with chronic stress (Redondo-Flórez et al., 2022). Regular sleep and eating patterns serve as fundamental physiological regulators that maintain homeostasis, stabilize mood, and optimize cognitive function. A disruption of these patterns impairs these processes and reduces an individual’s academic performance (Suardiaz-Muro et al., 2023). Consequently, poor self-regulation emerges not only as a correlate but also as a probable causal pathway to burnout, highlighting the importance of university-led wellness programs that prioritize basic lifestyle management. To operationalize these protective factors, stakeholders could consider implementing a comprehensive ‘Sleep Hygiene Program’. This program could be designed to educate students on the benefits of regular sleep, provide resources such as campus sleep clinics or workshops on time management, and create sleep-friendly environments by regulating study hours and ensuring dormitories are conducive to restful sleep.

The stepwise, dramatic increase in the odds of AB with declining academic class ranking is particularly revealing. It demonstrates that AB is not a binary state affecting only “failing” students but exists on a continuum heavily influenced by perceived academic standing. This gradient likely reflects the internalization of a competitive, rank-based evaluative culture. Students with lower rankings may experience chronic threats to their academic self-efficacy and future career prospects, which can fuel exhaustion and cynicism. Amid increasing educational competition and a constrained job market, academic performance has emerged as a central source of psychological distress for university students, and elevated academic stress readily triggers or aggravates AB (Sun et al., 2025).

The higher prevalence of AB among lower-year students challenges the common assumption that stress accumulates linearly. This “first-year peak” may be attributed to acute adjustment stress, the increased academic demands, adapting to university life, and building new social networks (Chen et al., 2021). Upper-year students, while facing heavier academic loads, may have developed more effective coping strategies, specialized study skills, and stronger peer support networks (Wu and Dong, 2024). This pattern suggests that targeted orientation and support are most critical at the transition into university life.

### Factors associated with academic burnout

The strong association between religious affiliation and an increased risk of AB is particularly noteworthy and warrants cautious, context-specific interpretation. In this study, students with religious affiliation (3.0% of the sample) had a markedly higher prevalence of AB (55.00%) compared to those without (19.81%), with an adjusted odds ratio of 5.56 (95% CI, 1.93–15.87). Given that the vast majority (97%) of participants reported no religious affiliation, belonging to a religious minority in this secular campus environment likely contributes to minority stress. Therefore, the elevated burnout risk is not attributable to religiosity itself but rather to the chronic strain of navigating a setting where one’s beliefs are uncommon. Potential mechanisms include: (a) time conflicts between religious practices and demanding medical schedules; (b) lack of understanding from peers and faculty; (c) difficulties in reconciling personal beliefs with the secular and demanding environment of medical training; and (d) absence of spiritual support services on campus (Sreeramareddy et al., 2007; Radcliffe and Lester, 2003; Popa-Velea et al., 2017). Our finding highlights the importance of fostering an inclusive campus climate that supports diverse worldviews.

Our findings strongly confirm the foundational role of health behaviors. An irregular daily routine emerged as a major modifiable risk factor, doubling the odds of AB. This aligns with the conservation of resources theory, which suggests that circadian and routine dysregulation depletes physiological and psychological resources, impairing stress recovery and cognitive function, thereby creating a vicious cycle with academic demands (Hobfoll, 1989; Leroy, 2009). This underscores that interventions targeting time management and sleep hygiene are not peripheral but central to preventing AB.

The gradient relationship between academic ranking and AB is a striking and critical finding. The stepwise increase in odds from the top 5% to the bottom 20% suggests that the culture of academic ranking itself, along with associated perceptions of self-efficacy and peer comparison, is a potent systemic stressor (Jia and Yeung, 2023; Gao, 2023; Pololi et al., 2012). For TCM students, this pressure is compounded by the need to excel in two vast, distinct knowledge systems: TCM (rooted in classical philosophy, pattern differentiation, and holistic principles) and Western biomedicine (grounded in anatomy, physiology, and evidence-based mechanisms). The unique stressors arising from this dual curriculum include: (a) cognitive overload from learning two incompatible conceptual frameworks; (b) methodological tension between the empirical-hermeneutic approach of TCM and the reductionist-experimental approach of modern medicine; (c) time allocation dilemmas with limited hours to master both; and (d) assessment anxiety due to different evaluation criteria (Sun et al., 2025; Zhao et al., 2025; Nie, 2014). These factors create a qualitatively distinct stress profile for TCM students, contributing to elevated burnout risk.

Finally, and most decisively, perceived teacher support (TS) stood out as the strongest protective factor. Each unit increase in the TS score was associated with a 22% decrease in the odds of AB. School climate refers to the internal psychological characteristics shared by school members, including teacher-student interpersonal relationships, behavioral norms, and feelings of belonging (Bradshaw et al., 2014). In the context of TCM education, which traditionally emphasizes the master-apprentice (“Shi Tu”) relationship, the teacher’s role extends beyond knowledge transmission to modeling clinical demeanor and professional identity. Supportive teachers can make the dual-curriculum challenge feel guided rather than overwhelming, directly mitigating emotional exhaustion and cynicism while fostering a sense of efficacy (Dandan et al., 2025).

### Implications for theory and practice

Our findings provide actionable guidance for educational stakeholders. Rather than advocating uniform approaches to burnout prevention, we propose targeted interventions across multiple domains: First, professional development for educators should emphasize not only pedagogical skills but also the ability to identify student distress, deliver growth-oriented feedback, and foster inclusive instructional environments characterized by supportive mentorship. Our data indicates that educational relationship-building represents the most impactful intervention point. Second, academic institutions need to formalize systematic training in self-regulatory capabilities, encompassing time management protocols, sleep optimization practices, and daily routine stabilization techniques. Consideration should be given to positioning such skill-building workshops or required courses as fundamental components of the core curriculum rather than optional supplements. Third, student support services, such as academic advising and psychological counseling, should enhance early identification and proactive outreach to vulnerable subgroups, including students from urban backgrounds, those with lower academic performance, and individuals from religious minority groups. Additionally, cross-year peer mentorship initiatives may also facilitate the normalization of academic challenges and the exchange of contextually relevant coping strategies. Finally, while academic ranking may remain necessary, institutions should implement comprehensive academic support structures to address students’ needs across the performance spectrum and minimize stigmatization.

### Limitations and strengths

This study possesses several notable strengths that enhance the credibility and value of its findings. Notably, the study addresses a significant gap in existing literature by focusing specifically on undergraduate populations within TCM educational settings. This cohort has received relatively little attention compared to medical students in Western education systems. Methodologically, this study employs a multi-tiered ecological conceptual framework that transcends isolated factor analysis by simultaneously examining interactions between individual characteristics, family background, and school climate. This integrative methodological approach yields a more nuanced, comprehensive perspective on the multifaceted origins of academic burnout in this unique educational context. Furthermore, the investigation implemented rigorous methodological standards: the sample size was not only substantial but also exceeded the minimum requirements determined by statistical analysis. At the same time, the use of psychometrically validated assessment instruments (MBI-SS, PSCS)-which demonstrated strong reliability coefficients in our participant group-further strengthens the empirical foundation of the findings.

Several limitations also should warrant consideration. First, the cross-sectional design precludes causal inference. While we hypothesize that low teacher support leads to AB, reverse causality-where AB colors perceptions of support- is possible. Second, the sampling strategy, which relied on convenience sampling from a single prestigious TCM institution, limits the generalizability of the findings to the broader TCM student population. Third, reliance on self-reported data for sensitive topics like mental health history or AB may be subject to social desirability bias. Finally, the analytical framework did not incorporate assessment of potentially relevant psychological constructs, such as resilience, perfectionism, or specific coping strategies, which might function as mediating or moderating variables within the observed relationships.

Future research in this domain should pursue several methodological advancements. Longitudinal designs would enable more precise delineation of causal pathways and tracking of burnout trajectories throughout the TCM educational journey. Qualitative studies are urgently needed to explore the lived experiences of the high-risk groups identified here (e.g., religious students, urban students) and to understand the mechanisms underlying the statistical associations. Finally, intervention studies are also essential to evaluate the effectiveness of targeted programs designed to strengthen teachers’ supportive capacities and enhance students’ self-regulatory skills, with AB metrics serving as primary outcome measures.

## Conclusion

This study extends beyond documenting the prevalence of AB among TCM students by elucidating its complex, multifactorial determinants. Our findings reveal that burnout results from the interaction of pre-existing demographic factors (such as urban residence and religious affiliation), modifiable behavioral patterns (including daily schedule regularity), systemic institutional pressures (notably academic ranking), and critical environmental resources that can be adjusted (particularly teacher support). These results reframe the discourse around AB, shifting from an individual-deficit perspective to a recognition of shared responsibility across the educational ecosystem.

Based on these findings, we propose four actionable strategies for TCM educational leaders and faculty: (a) training faculty to provide supportive mentorship and growth-oriented feedback, thereby strengthening teacher-student relationships; (b) integrating mandatory courses on time management and sleep hygiene into the core curriculum to promote regular daily routines; (c) implementing targeted support programs for students with lower academic rankings and those from religious minority backgrounds, addressing their specific vulnerabilities; and (d) establishing cross-year peer mentoring initiatives to facilitate the exchange of coping strategies and social support. Collectively, these evidence-based interventions aim to foster supportive learning environments and equip students with essential self-management skills, thereby safeguarding the psychological well-being and professional development of future TCM practitioners.

## Data Availability

The original contributions presented in the study are included in the article/Supplementary material, further inquiries can be directed to the corresponding authors.

## Footnotes

1
https://www.wjx.cn/
