# Supplementary material for: Prevalence and determinants of academic burnout among undergraduates in a traditional Chinese medicine university: a cross-sectional study
Source: Front Psychol. 2026 Jun 17;17:1799611. doi: 10.3389/fpsyg.2026.1799611 (PMC13319021; doi:10.3389/fpsyg.2026.1799611)
Supplement: Supplementary file 1 [file Supplementary_file_1.docx]

**sTable 1 Descriptive statistics of MBI-SS and PSCS with internal consistency**

| Variables | Cronbach's Alpha^＃^ | Bartlett's Test of Sphericity | | KMO measure of sampling adequacy |
| --- | --- | --- | --- | --- |
|  |  | χ² | *P* |  |
| EE | 0.833 | 1205.001 | ＜0.001 | 0.824 |
| CY | 0.768 | 837.420 | ＜0.001 | 0.662 |
| AE | 0.854 | 1523.089 | ＜0.001 | 0.886 |
| MBI-SS | 0.747 | 4454.232 | ＜0.001 | 0.887 |
| TS | 0.879 | 2549.624 | ＜0.001 | 0.854 |
| SSS | 0.867 | 4365.802 | ＜0.001 | 0.866 |
| OFA | 0.897 | 1911.952 | ＜0.001 | 0.887 |
| PSCS | 0.910 | 9565.098 | ＜0.001 | 0.907 |

Note: AE, academic efficacy; CY, Cynicism; EE, emotional exhaustion; MBI-SS, Maslach Burnout Inventory-Student Survey; OFA, opportunities for autonomy; PSCS, perceived school climate scale; SSS, student - student support; TS, teacher support.

^＃^ Cronbach’s alpha evaluated internal consistency and a value > 0.70 was considered acceptable.
